# Growth of *Pseudomonas taiwanensis* VLB120∆C biofilms in the presence of *n*‐butanol

**DOI:** 10.1111/1751-7915.12413

**Published:** 2016-10-03

**Authors:** Babu Halan, Igor Vassilev, Karsten Lang, Andreas Schmid, Katja Buehler

**Affiliations:** ^1^ Department of Solar MaterialsHelmholtz‐Centre for Environmental Research – UFZ GmbHPermoserstraße 1504318LeipzigGermany; ^2^ Laboratory of Chemical BiotechnologyDepartment of Biochemical and Chemical EngineeringTU Dortmund UniversityEmil‐Figge‐Str. 6644227DortmundGermany

**Keywords:** biofilms, butanol, solvent tolerance, adaptation, biocatalysis

## Abstract

Biocatalytic processes often encounter problems due to toxic reactants and products, which reduce biocatalyst viability. Thus, robust organisms capable of tolerating or adapting towards such compounds are of high importance. This study systematically investigated the physiological response of *Pseudomonas taiwanensis* VLB120∆C biofilms when exposed to *n*‐butanol, one of the potential next generation biofuels as well as a toxic substance using microscopic and biochemical methods. Initially *P. taiwanensis* VLB120∆C biofilms did not show any observable growth in the presence of 3% butanol. Prolonged cultivation of 10 days led to biofilm adaptation, glucose and oxygen uptake doubled and consequently it was possible to quantify biomass. Complementing the medium with yeast extract and presumably reducing the metabolic burden caused by butanol exposure further increased the biomass yield. In course of cultivation cells reduced their size in the presence of *n*‐butanol which results in an enlarged surface‐to‐volume ratio and thus increased nutrient uptake. Finally, biofilm enhanced its extracellular polymeric substances (EPS) production when exposed to *n*‐butanol. The predominant response of these biofilms under *n*‐butanol stress are higher energy demand, increased biomass yield upon medium complements, larger surface‐to‐volume ratio and enhanced EPS production. Although we observed a distinct increase in biomass in the presence of 3% butanol it was not possible to cultivate *P. taiwanensis* VLB120∆C biofilms at higher *n*‐butanol concentrations. Thereby this study shows that biofilms are not per se tolerant against solvents, and need to adapt to toxic *n*‐butanol concentrations.

## Introduction

A major challenge in many whole‐cell based biocatalytic production processes is the toxicity of educts as well as target products for the biocatalyst. For example most of the current biofuel production processes are economically not feasible because of end‐product inhibition. Due to the necessity of higher titres, several studies have focused on pushing the tolerance limit of the respective strains by rational engineering. Towards this goal, understanding of tolerance or adaptation mechanisms of the microorganism when challenged with the target compound is highly important (Dunlop, 2011). Specialized bacteria respond to solvents by several adaptation strategies, including expression of solvent extrusion efflux pumps, induction of heat‐shock proteins, membrane modification, vesicle formation and activation of general stress response genes (Isken and de Bont, 1998; Dunlop, 2011; Segura *et al*., 2012; Ramos *et al*., 2015). In addition, technical solutions like in situ product recovery for keeping the internal product concentration low and optimization of growth conditions are also applied (Heipieper *et al*., 2007; Rühl *et al*., 2009; Dunlop, 2011; Volmer *et al*., 2014).

In natural systems biofilms are known for enhanced robustness compared with their planktonic counterparts (Keweloh *et al*., 1989; Beveridge *et al*., 1997). These microbial communities are encased in self‐produced viscoelastic extracellular polymeric substances (EPS), which is highly hydrated and composed of polysaccharides, proteins, lipids, nucleic acids and other macromolecules produced by the cells within (Flemming and Wingender, 2010). Besides being a major part of a biofilm, EPS production can be regarded as a natural response upon exposure to a toxic environment (Fang *et al*., 2002; Sheng *et al*., 2005, 2010; Van Acker *et al*., 2014). In general, biofilms respond to stress in many different ways e.g. reducing their growth rate or by simply leaving the community via detachment (Spormann, 2008). Also a transition from exponential to slow or no growth of biofilms was observed upon exposure to antibiotic stress (Mah and O'Toole, 2001; Sutherland, 2001; Stewart and Franklin, 2008).

Various stress factors, especially antimicrobial substances, have been studied in the context of biofilm tolerance. Regarding catalytic biofilms which have been shown to be potent biocatalysts especially for the conversion of toxic compounds (Halan *et al*., 2012), looking at stress related behaviour is highly interesting, as this may have major impact on their catalytic performance. Only little is published in this respect, aside from a study looking at the influence of styrene and (*S*)‐styrene oxide on biofilm productivity and integrity of *Pseudomonas taiwanensis* VLB120ΔC biofilms. Although heavily permeabilized upon solvent addition, these cells recovered, enhanced the production of polysaccharides, and apparently adapted to the toxic conditions (Halan *et al*., 2011). To further extend our knowledge and explore the suitability of catalytic biofilms as cell factories for the synthesis of *n*‐butanol (referred to as butanol hereafter) the present work is dedicated to elucidate the general biofilm response when challenged with this compound. Butanol is a potential next generation biofuel known to severely reduce cellular vitality (logP_o/w_: 0.8; partition coefficient of a compound in an equimolar mixture of octanol and water) most probably by interfering with the hydrogen bonds of membrane phospholipids (Neumann *et al*., 2005a, b). Unlike conventional fossil fuels, next generation biofuels are considered to be more sustainable as feedstocks and have reduced green‐house gas emissions during production steps. Most importantly, they do not compete with food crops for land use. Moreover, next generation biofuels should be compatible with existing infrastructure i.e. storage and transportation (www.biofuelstp.eu). So far, only a limited number of exclusively planktonically growing bacteria have been reported to tolerate a maximum of 2–3% (v/v) butanol (Dunlop, 2011). *P. taiwanensis* VLB120 is able to grow on styrene as sole carbon and energy source. The here used variant *P. taiwanensis* VLB120∆C carries an insertion in the isomerase encoding gene disrupting the styrene degradation pathway for accumulation of styrene oxide. The styrene degradation pathway has no influence on the ability of the strain to cope with butanol and aside from this mutation both strains do not differ in their genome sequence. Biofilms of the variant VLB120∆C have been extensively applied for various biocatalytic reactions already (Halan *et al*., 2012, 2014; Gross *et al*., 2013). Therefore, we selected this particular variant for this study and elucidated the influence of butanol on biomass yield, oxygen and glucose uptake, cellular morphology and the EPS profile using a combination of biochemical assays, as well as CLSM and SEM based imaging.

## Results

### Biofilm biomass yield of *P. taiwanensis* VLB120∆C depends on butanol concentration in the medium

**Figure 1:**
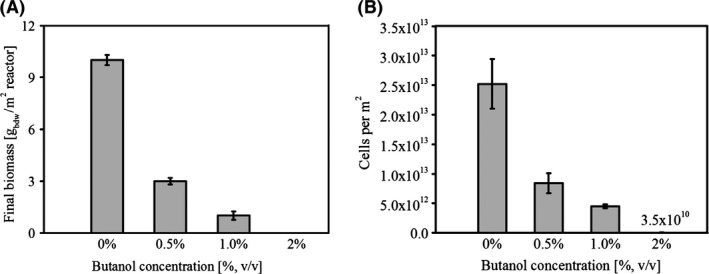
Physiological response and butanol concentration dependent biomass yield of *P. taiwanensis* VLB120∆C biofilms. (A) Biofilm growth expressed as the amount of biomass produced on a given reactor surface in the presence of different butanol concentrations. (B) Cell numbers per square metre of the reactor surface. Biofilm was cultivated for 2 days. Data presented here are mean values from four parallel experiments.

There are basically two strategies to analyse the solvent tolerance reported in literature. Either the organism is grown from the start in the presence of the respective solvent, which allows investigation of long‐term solvent response mechanisms or a solvent shock is applied to already grown cultures revealing short‐term solvent responses (Cuenca *et al*., 2016a). In this study, we focused on the long‐term adaptation to *n*‐butanol. To investigate biofilm growth and biomass yield at different butanol concentrations, growth experiments were performed in defined minimal medium. Figure 1A shows the total biomass attaching to the available growth surface in g_bdw_/m^2^_growth surface_ quantified by drying the biofilm at the end of day two. Final biofilm biomass yield and the cell numbers (Fig. 1) decreased gradually, correlating with increasing butanol concentrations in the medium. Compared with the control without butanol, the final biomass was reduced by more than 50% at the lowest butanol concentration applied. At 2% (v/v) butanol no growth was visible by eye, but a significant amount of cells could still be detected and counted by microscopic analysis (Fig. 1B). PI staining revealed that these were consisting of both intact and permeabilized cells, however, with rising butanol concentrations the number of intact cells was drastically reduced (data not shown). Cells were also cultivated planktonically at different butanol concentration in shake flasks in order to compare them with the biofilm grown counterparts. Already 0.5% butanol had a significant impact on the biomass yield as it was reduced by nearly 45% whereas 1% butanol concentration lowered the biomass yield by more than 90% as compared with the control without any butanol (Fig. S4).

In addition, biofilm experiments have been conducted where a solvent shock was applied to a 3 days old biofilm of *P. taiwanensis* VLB120. Subsequently, the survival rates were determined and expressed as colony forming units (CFUs/mL, Figs S2 and S3). Findings have been similar to the long‐term treatment.

**Figure 2:**
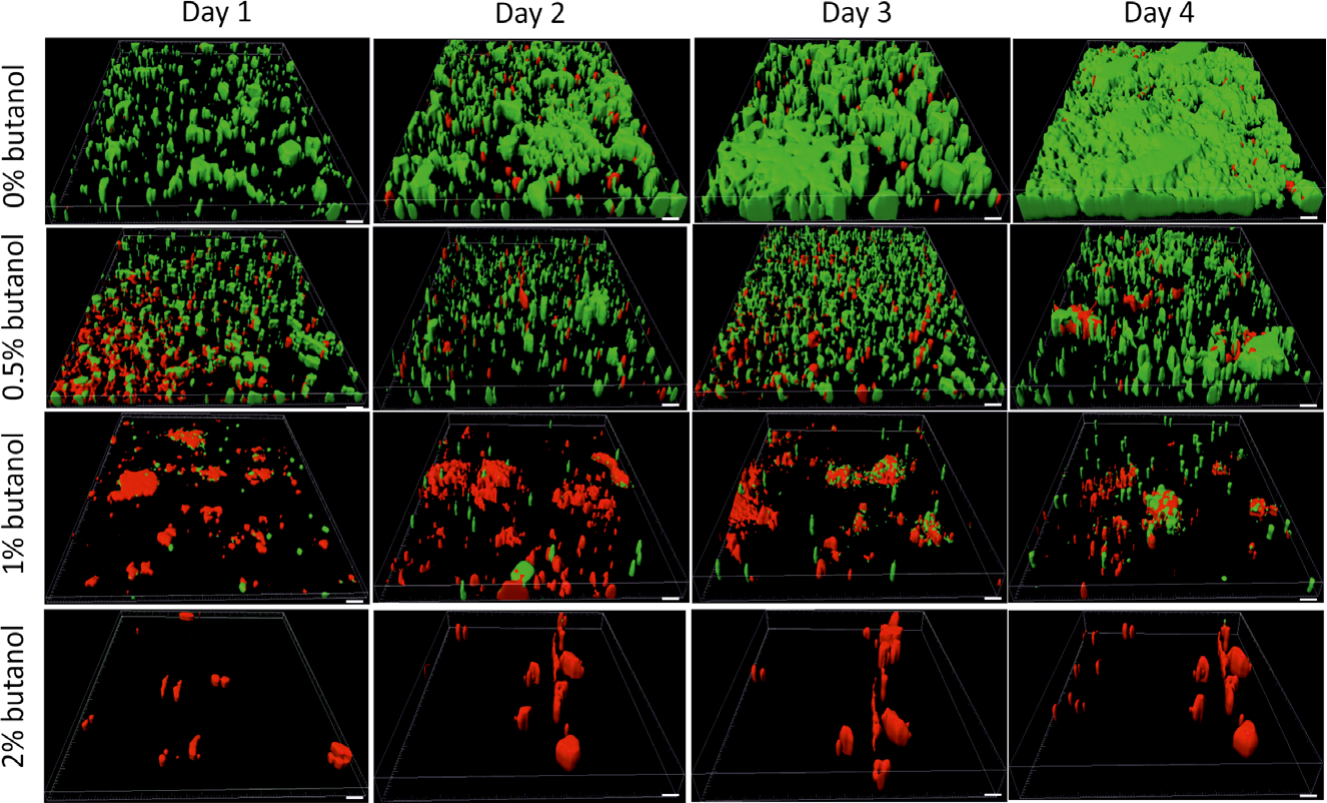
Micrographs showing biofilm development of *P. taiwanensis* VLB120∆C*egfp* under standard growth conditions (no butanol) and in the presence of increasing butanol concentrations in minimal medium. Green colour represents the intact *gfp*‐expressing cells; red colour represents PI‐stained dead and/or permeabilized cells. Representative IMARIS‐treated and 3D‐reconstructed images from three parallel experiments are shown. Scale bar, 20 μm.

To support the data obtained from the growth experiments performed in a tubular membrane reactor, biofilms were cultivated in a flow‐cell under the same conditions and examined by CLSM (Fig. 2). In a butanol free environment, micro‐colonies were already observed at the first day of cultivation and after 4 days the whole surface was covered by cells (Fig. 2). As the butanol concentration increased less surface was covered. In addition, the fraction of permeabilized cells multiplied, until at 2% butanol barely any intact cells could be detected. From these results it can be concluded that butanol had a severe impact on biofilm growth and integrity and already at 0.5% butanol a strong impact of this compound was observed. These results are consistent with the outcome of the growth experiments performed in the silicone tubing described above.

### The presence of butanol increased the energy requirement of *P. taiwanensis* VLB120∆C biofilms

**Figure 3:**
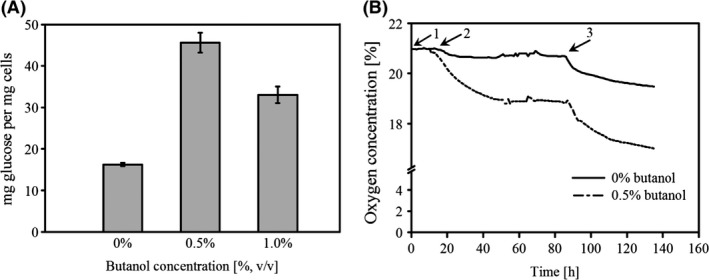
Consumption of glucose and oxygen by *P. taiwanensis* VLB120∆C grown with and without butanol in the medium for 2 days. (A) Glucose consumption. (B) Oxygen consumption. 1 ‐ Medium flow before inoculation, 2 ‐ Inoculated system under continuous flow (100 μL min^−1^), 3 ‐ Medium flow stopped, system completely closed. Data presented here are mean value from two different experiments.

Glucose and oxygen consumption by the biofilm was monitored as readout for the energy requirement of the organism. Biofilms grown in the presence of butanol consumed more than double the amount of glucose (biomass yield on glucose Y_x/s_ = 0.022) compared with the biofilms grown without any butanol (Y_x/s_ = 0.062; Fig. 3A). Strikingly, at 1% butanol the amount of glucose consumed per biomass was less as compared with 0.5%. This is probably due to the rising number of dead cells which are still contributing to the overall biomass although not consuming glucose anymore (Fig. 2). In addition, the oxygen consumption rate increased significantly upon solvent addition (Fig. 3B). It attained a steady state approximately 50 h after the cultivation was started. It was assumed, that at this time point the oxygen present in the headspace of the reaction system was in equilibrium with the oxygen dissolved in the medium continuously running through the tubing. In order to quantify the available oxygen in the medium, the medium flow was stopped by clamping the reactor inlet and outlet. As can be seen in Fig. 3B, the oxygen consumption by the biofilm in the presence of butanol was significantly higher even though threefold less biomass was formed. Since the biofilm barely grew in the presence of butanol the increased glucose as well as oxygen consumption may be attributed to a greater energy demand for maintenance of the cellular functions.

### Butanol alters the cellular morphology of *P. taiwanensis* VLB120∆C

**Figure 4:**
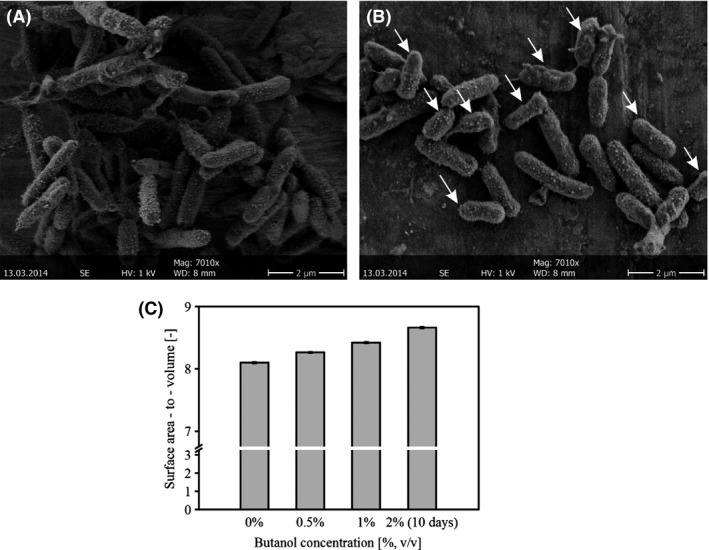
Cell size of *P. taiwanensis* VLB120∆C biofilm grown with and without butanol. (A and B) SEM micrographs of the cellular morphology without butanol (A) and with 0.5% butanol (B). Arrows indicate the smaller cells. (C) Surface area‐to‐volume ratio of the cells grown at different butanol concentration for 2 and 10 days. Data presented here are mean value from two different experiments. The size of approximately 100 cells from each culture was measured.

To investigate the morphological changes of the cells upon butanol exposure, *P. taiwanensis* VLB120∆C*egfp* biofilms were analysed using CLSM and SEM. Based on the SEM micrographs the cell size was calculated. Compared with the control without butanol, the average cell size decreased in the presence of butanol (Fig. 4A and B). In a butanol free environment, cells had an average length of 2.2 ± 0.14 μm compared with 1.28 ± 0.27 μm when grown in 0.5% butanol. In addition, a gradual decrease in cell length was observed with increasing butanol concentration. Reduction in cell size resulted in a rise in the relative cell surface with respect to the volume (Fig. 4C), which might have facilitated the enhanced glucose and oxygen uptake. This phenomenon was discussed for planktonically growing *P. putida* P8 cells which were able to alter their size upon exposure to toxic chemicals (Neumann *et al*., 2005b). Our results indicate that cells growing in biofilms can also show morphological response towards toxic chemical like butanol.

### Butanol triggers EPS production in *P. taiwanensis* VLB120∆C biofilms

**Figure 5:**
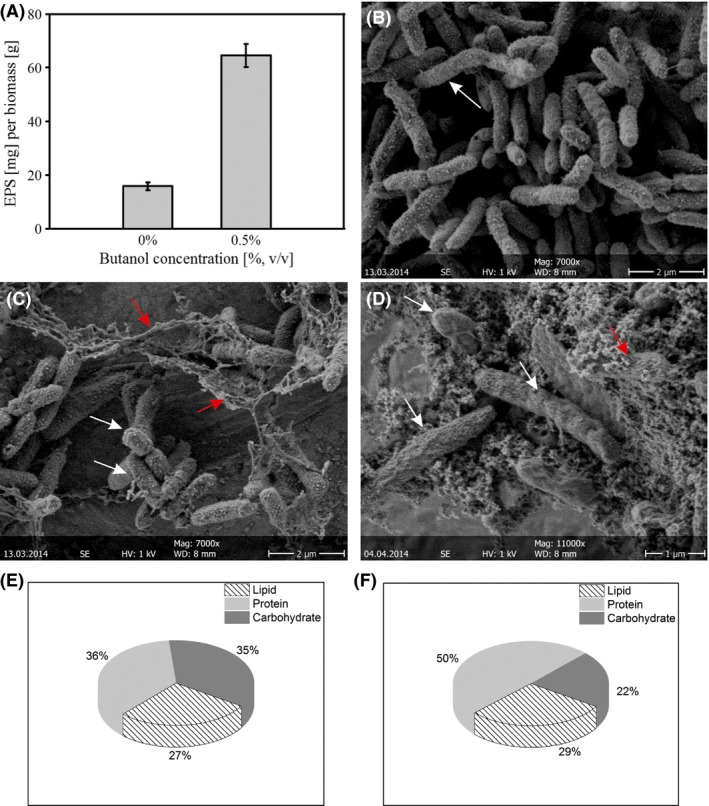
EPS profile of the biofilm grown with and without butanol. (A) Total amount of EPS produced by biofilms of *P. taiwanensis* VLB120∆C under standard conditions and in the presence of butanol. (B) SEM images of the dehydrated biofilm grown without butanol. (C) With 0.5% butanol. (D) With 2% butanol for 10 days. White arrows show the cells and red arrows show the denatured EPS. (E and F) Main EPS compounds detected in the EPS of biofilms grown under standard conditions and in the presence of 0.5% butanol. Data presented here in (A, E and F) are mean value from four different experiments.

EPS play a major role for the structural integrity of biofilms. In the present system, total EPS production was monitored and subsequently quantified in a butanol free environment, as well as in the presence of 0.5% butanol. Overall it was observed that EPS production was doubled when biofilms were cultivated in the presence of butanol (Fig. 5A) and the ratio of the single EPS compounds, especially proteins and carbohydrates, changed dramatically (Fig. 5E and F). Proteins and carbohydrates each accounted for approximately 35% of the EPS in biofilms cultivated under standard (no solvent) conditions. Upon solvent addition, the fraction of the proteins increased to 50% while carbohydrates decreased to 22%. In addition to the calorimetric methods, EPS was qualitatively investigated using SEM (Fig. 5B–D). Abundant dehydrated and structurally altered EPS could be visualized in the biofilm grown in 0.5% butanol which significantly increased in the presence of 2% butanol (Fig. 5C and D).

### Medium engineering and prolonged cultivation time enable biofilm formation of *P. taiwanensis* VLB120∆C up to 3% butanol

**Figure 6:**
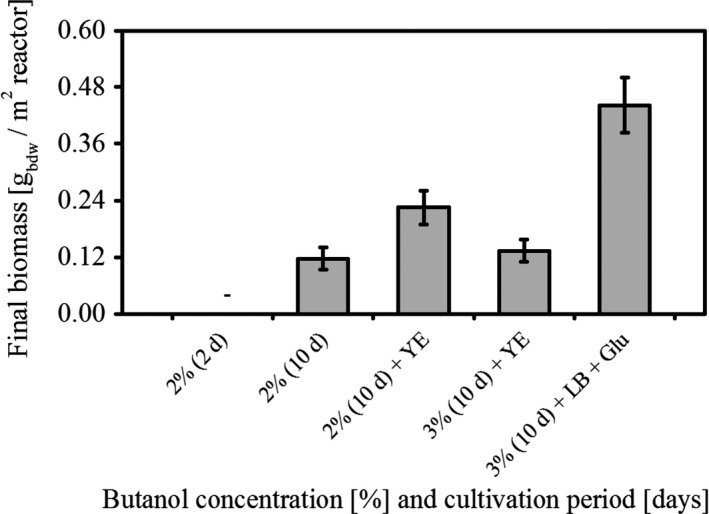
Medium dependent biofilm growth of *P. taiwanensis* VLB120∆C at increasing butanol concentrations. Data presented here are mean values from three different experiments.

To study the maximum possible butanol concentration in which *P. taiwanensis* VLB120∆C biofilms grow, a systematic growth experiment was conducted. The impact of a prolonged cultivation and adaptation period was evaluated for biofilm growth in the presence of 2% and 3% butanol. Interestingly, the organisms obviously adapted to the harsh conditions over time and started to grow, although slowly and not reaching 100% surface coverage (Fig. 6). Biomass nearly doubled when the medium was supplemented with 0.5% (w/v) yeast extract. Addition of yeast extract not only enhanced the biomass but also surpassed the growth inhibitory limit of 2% butanol as quantifiable biofilm growth could now also be detected at 3% butanol. Finally, the biomass yield increased further when the biofilm was grown in a complex LB medium containing 3% butanol. This behaviour suggests that medium supplementation can ease the toxicity burden by decreasing the cost for sustaining biomass synthesis through the direct supply of e.g. amino acids and other precursors.

## Discussion

Toxicity is one of the major barriers for commercial scale production of biofuels and other value added chemicals. Butanol as a next generation biofuel is well‐known in this context and significantly reduces cellular vitality, a critical issue in practical bio‐butanol production (Kanno *et al*., 2013). Prior studies have documented various microbial strains that are capable of thriving at elevated butanol concentrations. Amongst the Pseudomonads there are various strains reported, which are capable to cope with elevated butanol concentrations. *P. taiwanensis* VLB120 was characterized previously for its butanol tolerance (Rühl *et al*., 2009). It was reported that after repeated adaptation in a butanol environment, the strain was able to grow in the presence of up to 6% (vol/vol) butanol added (4% (vol/vol) measured) in planktonic culture while the non‐adapted cells could withstand butanol up to 3% (Rühl *et al*., 2009). It was not possible to reproduce these results in long‐term growth experiments. Although cells seem not to lyse at elevated butanol concentrations suggesting survival, sustainable cell growth was heavily impaired already at 1% (vol/vol) butanol added (data not shown). The results obtained in our study also reflected that the non‐adapted planktonically grown *P. taiwanensis* VLB120∆C cells are highly susceptible to 1% butanol (Fig. S4).

In addition *P. putida* DOT‐T1E, *P. putida* KT2440 and *P. putida* BIRD‐1 have been studied in detail regarding their ability to thrive in the presence of higher butanol concentrations (Ramos *et al*., 2015; Cuenca *et al*., 2016a, b). The doubling time of BIRD‐1 at 0.8% butanol was significantly higher compared with KT2440 and DOT‐T1E. In addition, BIRD‐1 did not show any significant decrease in viability up to 2% butanol whereas the viability of other strains decreased significantly in this parameter (Cuenca *et al*., 2016a, b). In searching for other butanol tolerant microbes, gram positive *Lactobacillus* strains, *L. delbrueckii* and *L. brevis*, were found to tolerate and grow as planktonic culture in a complex medium containing up to 3% butanol (Knoshaug and Zhang, 2009; Liu and Qureshi, 2009). For a new isolate CM4A, belonging to genus *Enterococcus*, even 3.5% butanol in a medium supplemented with yeast extract and tryptone have been reported (Kanno *et al*., 2013). This strain exhibited increased membrane fatty acid saturation to maintain the fluidity as a response to butanol exposure (Kanno *et al*., 2013). Butanol is highly soluble in cellular membranes (up to 1.59 M). It intercalates in the membrane and thereby the hydrogen bonds between the lipid tails are broken, leading to changes in the fatty acid composition. This is reflected by an increase in membrane fluidity (Huffer *et al*., 2011; Neumann *et al*., 2005a, b; Rühl *et al*., 2012). Understanding the response or adaptation mechanisms of the biocatalyst when exposed to toxic and process relevant solvents is essential to exploit the biocatalyst and design robust bioprocesses. Various adaptive mechanisms have been identified at different levels.

Solvent‐tolerant *Pseudomonas* strains (e.g. *P. putida* S12, *P. putida* DOT‐TIE, *P. putida* BIRD‐1) use multifactorial response mechanisms that involve changes in gene expression, modification of membrane fluidity, induction of chaperones or heat shock proteins, activation of oxidative stress response, and enhanced energy generation when exposed to toxic solvents. Some bacteria also employ specific efflux pumps to extrude toxic compounds (Segura *et al*., 2012; Ramos *et al*., 2015; Simon *et al*., 2015; Vallon *et al*., 2015).

The idea of this study was to evaluate if the biofilm mode of growth, which in general is attributed to increased tolerance against various kind of environmental stress factors is also increasing butanol tolerance in *P. taiwanensis* VLB120∆C, a strain known for its ability to convert alkanes and styrene derivatives (Park *et al*., 2007; Gross *et al*., 2013; Halan *et al*., 2014). *P. taiwanensis* VLB120∆C showed a couple of responses towards butanol addition. Apart from a considerable decrease in growth and biomass formation it required more glucose and oxygen than under non‐solvent conditions. These data support the hypothesis that solvent tolerance is an energy demanding process, which was reported for a couple of organisms growing in planktonic cultures (Huffer *et al*., 2011). For example, in *P. putida* DOT‐T1E, *P. putida* S12 and *E. coli* HG228, the genes of the TCA cycle involved in energy metabolism were induced in the presence of toxic solvents (Segura *et al*., 2012). The presence of solvents toluene, ethylbenzene, propylbenzene, xylene, hexane and cyclohexane in general decreased the biomass yield of *P. putida* S12 and increased the maintenance requirements. These effects are caused by energy‐consuming adaptation mechanisms (Isken *et al*., 1999). In the presence of butanol, the energy metabolism of *C. acetobutylicum* was also up‐regulated (Nicolaou *et al*., 2010). It is now widely accepted, that cells boost their energy metabolism in the presence of toxic solvents, which was also reported for a couple of other organisms as reviewed by Ramos *et al*. (2015).

Another adaptive response upon toxic solvent exposure is related to the alteration of the cellular morphology as reported for *P. putida* P8 and *Enterobacter* sp. VKGH12 (Neumann *et al*., 2005b). *P. putida* P8 cells increased in length when exposed to phenol and 4‐chlorophenol. The change in the morphology was explained by the relative reduction in cell surface with respect to its volume (Neumann *et al*., 2005b). The opposite was observed for *Enterobacter* sp. VKGH12 cells, which became shorter at higher butanol concentrations leading to an enhanced ratio between the surface and volume of the cells. However, this effect was observed only when butanol was used as sole carbon and energy source, whereas a contrary effect was perceived when the cells were grown in glucose containing medium supplemented with butanol as a sole toxin (Veeranagouda *et al*., 2006). Authors have argued that the relative reduction in attachable surface for toxic organic compounds to be the effective mechanism of the cells to reduce the toxic effect of environmental stress factors. In the latter case, better uptake and transformation of the butanol was considered to be the reason (Neumann *et al*., 2005b; Veeranagouda *et al*., 2006). However, information regarding nutrient uptake was not reported. *P. taiwanensis* VLB120∆C cells reduced their cell size upon butanol exposure leading to a higher ratio between the surface and volume of the cells. It was hypothesized that this increase in relative surface area enabled the cells to upsurge nutrient uptake. Elevated glucose and oxygen uptake rates supported this hypothesis.

In addition to the cellular morphology and energy metabolism, organisms growing in a biofilm respond to solvent stress by altering their EPS. Extracellular polymeric substances are primarily involved in physical adhesiveness, which plays a major role in biofilm formation, cell adhesion to solid surfaces and creation of protective microhabitats against a wide range of adverse environmental conditions (Dohnalkova *et al*., 2011). Enhanced EPS production is considered an important part of the stress response of the biofilm (Sutherland, 2001). This is in accordance to our findings regarding *P. taiwanensis* VLB120∆C biofilms which produced significantly more EPS upon butanol exposure (Fig. 5). Similar results were reported when styrene was used as solvent in *P. taiwanensis* VLB120∆C biofilm driven catalysis (Halan *et al*., 2011). Also the presence of toxic metals and salt stress has been shown to have a significant influence on EPS content and composition of bacterial biofilms (Fang *et al*., 2002; Sheng *et al*., 2005; Priester *et al*., 2006; Zhang *et al*., 2011), underlining the importance of EPS for biofilm stress tolerance.

*P. taiwanensis* VLB120∆C biofilm showed quantifiable growth up to 3% butanol in minimal medium supplemented with 0.5% yeast extract after a prolonged adaptation phase. In corresponding planktonic cultures under comparable cultivation conditions no growth could be detected (data not shown). Apart from the biofilm growth mode, also other intrinsic solvent adaptation mechanisms are known for this strain. It was shown, that the membrane composition of the *P. taiwanensis* VLB120 significantly altered upon butanol exposure (Rühl *et al*., 2012). In addition, a couple of solvent efflux pumps (TtgGHI and TtgABC) are present, enabling growth in the presence of toluene and styrene (Volmer *et al*., 2014).

### Concluding remarks

Biofilms of *P. taiwanensis* VLB120∆C are able to adapt to highly toxic solvents over a fairly long time period. The predominant response of these biofilms under butanol stress are higher energy demand, increased biomass yield upon medium complements, larger surface‐to‐volume ratio and enhanced EPS production. However, although it was possible to adapt *P. taiwanensis* VLB120∆C to toxic butanol concentrations it was not possible to cultivate the strain above 3% butanol, a value still below the maximum butanol tolerance capacity of 3.5% reported for planktonic cultures of *Enterococcus*. This clearly shows that biofilms are not per se highly tolerant against toxic substances as often reported in the literature and that the limits need to be investigated carefully for each compound.

## Experimental procedures

All chemicals used in this study were purchased either from Sigma‐Aldrich (Steinheim, Germany) or Carl Roth GmbH (Karlsruhe, Germany) unless stated otherwise. The chemicals were of the highest purity available and used without further purification. Luria‐Bertani medium (LB) was used for the pre‐cultures. M9 medium (Sambrook and Russell, 2001) supplemented with 0.5% (wt/vol) glucose as a carbon source, Uwe Sauer (US*) trace elements (Emmerling *et al*., 2002), and streptomycin (100 μg mL^−1^), was used for shake flask and reactor experiments.

### Pre‐culture cultivation

Pre‐cultures of *P. taiwanensis* VLB120 and *P. taiwanensis* VLB120∆C (Park *et al*., 2007) and *P. taiwanensis* VLB120∆C*egfp* (Halan *et al*., 2011) were grown overnight in 25 mL M9 medium (0.5% glucose) using baffled 250 mL Erlenmeyer flasks in a horizontal shaker (30°C and 200 r.p.m.; Multitron; Infors HT, Bottmingen, Switzerland).

### Cultivation of planktonic cell culture

Pre‐cultures of *P. taiwanensis* VLB120∆C have been used to inoculate the main culture with an initial biomass concentration of 0.09 g_cdw_ L^−1^ in 25 mL M9 medium (0.5% glucose) using screw‐cap baffled 250 mL Erlenmeyer flasks and subsequently respective butanol amount was added. The flasks were incubated in a horizontal shaker (30°C and 200 r.p.m.; Multitron; Infors HT, Bottmingen, Switzerland) and growth was monitored by analysing the final biomass density achieved. Only an end‐point measurement was performed to avoid butanol losses through the head‐space by sampling.

### Biofilm cultivation in flow‐cell systems

For microscopic analysis, a green fluorescent protein tagged variant of *P. taiwanensis* VLB120∆C was used, named *P. taiwanensis* VLB120∆C*egfp*. Respective biofilms were cultivated in a custom made flow‐cell system, which allowed real‐time fluorescence‐based optical analysis of cell physiology. Details of the flow‐cell setup are given in Halan *et al*., 2011. Medium transport occurred through a peristaltic pump (ISM 930; Ismatec, Wertheim‐Mondfeld, Germany). The assembled flow‐cell reactor was heat sterilized at 121°C for 20 min and flushed with sterile deionized water for 2–3 h. Subsequently, the deionized water was exchanged for M9 medium (0.5% glucose), and the system was conditioned for 2 h before being inoculated with an overnight culture of *P. taiwanensis* VLB120∆C*egfp* (4–5 mL, diluted to an optical density of 0.8–1.0 at 450 nm). Depending on the experiment, the medium was butanol saturated before inoculation. Butanol was added to the medium and pumped continuously through flow cell. During inoculation, the medium pump was stopped. After inoculation, the flow‐cell was kept idle without medium supply for 2–3 h to enable the initial attachment of the cells to the glass substratum. Medium feed was started with a flow rate of 70 μL min^−1^ (dilution rate, 7.0 h^−1^). The temperature in the flow‐cell was maintained at 30°C.

### Biofilm cultivation in tubular modules

For physiological analysis of biofilm and also for EPS production, *P. taiwanensis* VLB120ΔC was grown as biofilms on the inner surface of silicone tubing (Fig. S1). The system was inoculated with an over‐night grown culture of *P. taiwanensis* VLB120ΔC. A solvent resistant MASTERFLEX^®^ tube was used to connect the silicone tubes in both reservoirs to avoid any solvent losses during transport from the medium bottle. In the growth compartment, the silicone tube for biofilm growth was kept in the head space of the Schott bottle (1000 mL) as depicted in Fig. S1. For the initial attachment, the inoculated culture was kept 2 h without any medium flow. Subsequently, M9‐medium (0.5% glucose as carbon source) was supplied continuously using a peristaltic pump (ISM 930; Ismatec) at a flow rate of 100 μL min^−1^. Butanol supply to the biofilm was ensured by directly mixing it into the medium at different concentrations as indicated. As a control, two biofilm reactors were cultivated without butanol addition. At the end of each experiment, biomass was collected by mechanically disrupting the biofilm from the silicone walls and the resulting sludge was subjected to either biomass dry weight determination or EPS extraction followed by composition analysis as described in the following sections.

### Staining techniques

Propidium iodide (PI; Invitrogen, Eugene, OR, USA) was used to stain dead/permeabilized cells (excitation at 535 nm and emission at 617 nm). PI was mixed with the medium at 3 μM end concentration (according to the distributor's protocol) and pumped continuously through the flow cell.

### Image acquisition and data treatment

Image acquisition was performed using a Zeiss LSM5 Pascal confocal laser‐scanning microscope (CLSM; Carl Zeiss, Jena, Germany) equipped with an argon and helium‐neon laser. Images were obtained using an EC Plan‐Neofluar 20×, 0.50 Ph2M27 objective. Three‐dimensional image reconstructions, quantification of biofilms and living and dead cell distribution were done using the software package IMARIS (Bitplane AG, Zürich, Switzerland).

### Biomass dry weight analysis

Samples were collected from the silicone tube walls in a pre‐dried and weighted Falcon tube after 48 h of cultivation and dried at 85°C until constant dry weight. The biomass dry weight was normalized to the tube surface area as g_bdw_ m^−2^.

### Determination of colony forming units

The reactor outlet was collected continuously in sterile Falcon tubes cooled in an ice container. The number of CFU (CFU mL^−1^) was obtained by plating diluted aliquots of the collected samples on LB agar plates.

### EPS extraction

The biofilms were harvested after 48 h of cultivation. The EPS extraction method was based on Dowex resin as described in Liu and Fang (2002). Dowex resins were washed with 6 mM Potassium phosphate buffer prior to use. Briefly, 2 mL of the respective sample were mixed with 400 mg of pre‐washed Dowex resin at room temperature and 200 r.p.m.. Subsequently the samples were centrifuged at 20 000 *g* (Sorvall^®^ Discovery^™^ 90SE; Hitachi, Tokyo, Japan) for 20 min and the supernatant was filtered through a 0.22 μm membrane (MILLEX^®^GP 0.22 μm; Millipore, Darmstadt, Germany). To isolate low molecular‐weight EPS components, the solution was dialysed through a membrane with a cut‐off of 3500 Da (Cellu Sep F1; Size: 55 mm × 55 mm; MWCO: 3500; Thickness: 28 μm; Membrane Filtration Products, Seguin, TX, USA) at 4°C for 24 h. The sample was then stored at 4°C for further analysis.

### Analysis of EPS composition, butanol, glucose and oxygen concentrations

Different assays were established for quantifying EPS components grouped as proteins, carbohydrates, lipids and uronic acids (Table S1).

#### Glucose analysis

Aqueous samples from the reactor outlet were centrifuged (10 min; 4°C; 16 500 *g*; Thermo Fisher Scientific, Langenselbold, Germany) and the supernatant was used for the quantification of glucose using the Enzytec^tm^ 9 kit (R‐Biopharm AG, Darmstadt, Germany).

#### Oxygen concentrations BlueSens

Oxygen gas sensor BCP‐O_2ec_ (BlueSens gas sensor GmbH, Herten, Germany) was used to measure the oxygen concentration in the headspace of the reaction compartment. Calibration with fresh air was done according to the manufacturer's protocol.

#### Butanol quantification

Butanol was analysed and quantified using high pressure liquid chromatography equipped with UV‐Vis and RI detector (HPLC, LaChrom Elite, Merck Hitachi, Darmstadt, Germany) and a Trentec 308R‐Gel.H column (300 × 8 mm, Trentec Analysentechnik, Gerlingen, Germany) at 40°C. Five mM H_2_SO_4_ was used as mobile phase at a flow rate of 1.0 mL min^−1^.

## Conflicts of interest

None declared.

## Supporting information


**Fig. S1.** Schematic diagram of the tubular setup applied for biofilm biomass production.
**Fig. S2.** Survival of *P. taiwanensis* VLB120 grown in biofilms treated with different concentrations of butanol determined by colony forming units (CFU).
**Fig. S3.** Comparison of butanol concentration dependent biomass yield of *P. taiwanensis* VLB120 and mutant strain *P. taiwanensis* VLB120ΔC biofilms.
**Fig. S4.** Butanol concentration dependent biomass yield of planktonically grown *P. taiwanensis* VLB120 and *P. taiwanensis* VLB120ΔC.
**Table S1.** Methods for EPS component analysis.
