# Supplementary material for: Growth of Pseudomonas taiwanensis VLB120∆C biofilms in the presence of n‐butanol
Source: Microb Biotechnol. 2016 Oct 3;10(4):745–55. doi: 10.1111/1751-7915.12413 (PMC5481524; doi:10.1111/1751-7915.12413)
Supplement: Supplementary file 1 — Fig. S1. Schematic diagram of the tubular setup applied for biofilm biomass production. Fig. S2. Survival of P. taiwanensis VLB120 grown in biofilms treated with different concentrations of butanol determined by colony forming units (CFU). Fig. S3. Comparison of butanol concentration dependent biomass yield of P. taiwanensis VLB120 and mutant strain P. taiwanensis VLB120ΔC biofilms. Fig. S4. Butanol concentration dependent biomass yield of planktonically grown P. taiwanensis VLB120 and P. taiwanensis VLB120ΔC. Table S1. Methods for EPS component analysis. [file MBT2-10-745-s001.docx]

**Supporting information**

Growth of *Pseudomonas taiwanensis* VLB120∆C biofilms in the presence of *n*-butanol

Babu Halan^1^, Igor Vassilev^2^, Karsten Lang^2^, Andreas Schmid^1^, Katja Buehler^1,^*

* Corresponding author

1 Helmholtz-Centre for Environmental Research − UFZ GmbH

Department of Solar Materials

Permoserstraße 15, 04318 Leipzig, Germany

Tel: +49-341-235 46 83

Fax: +49-341-235 45 12 86

E-Mail: [katja.buehler@ufz.de](mailto:katja.buehler@ufz.de)

2 Laboratory of Chemical Biotechnology, Department of Biochemical and Chemical

Engineering, TU Dortmund University, Emil-Figge-Str. 66, 44227 Dortmund,

Germany

Tel: +49-231-7557381

Fax: +49-231-7557382

**Figure S1**


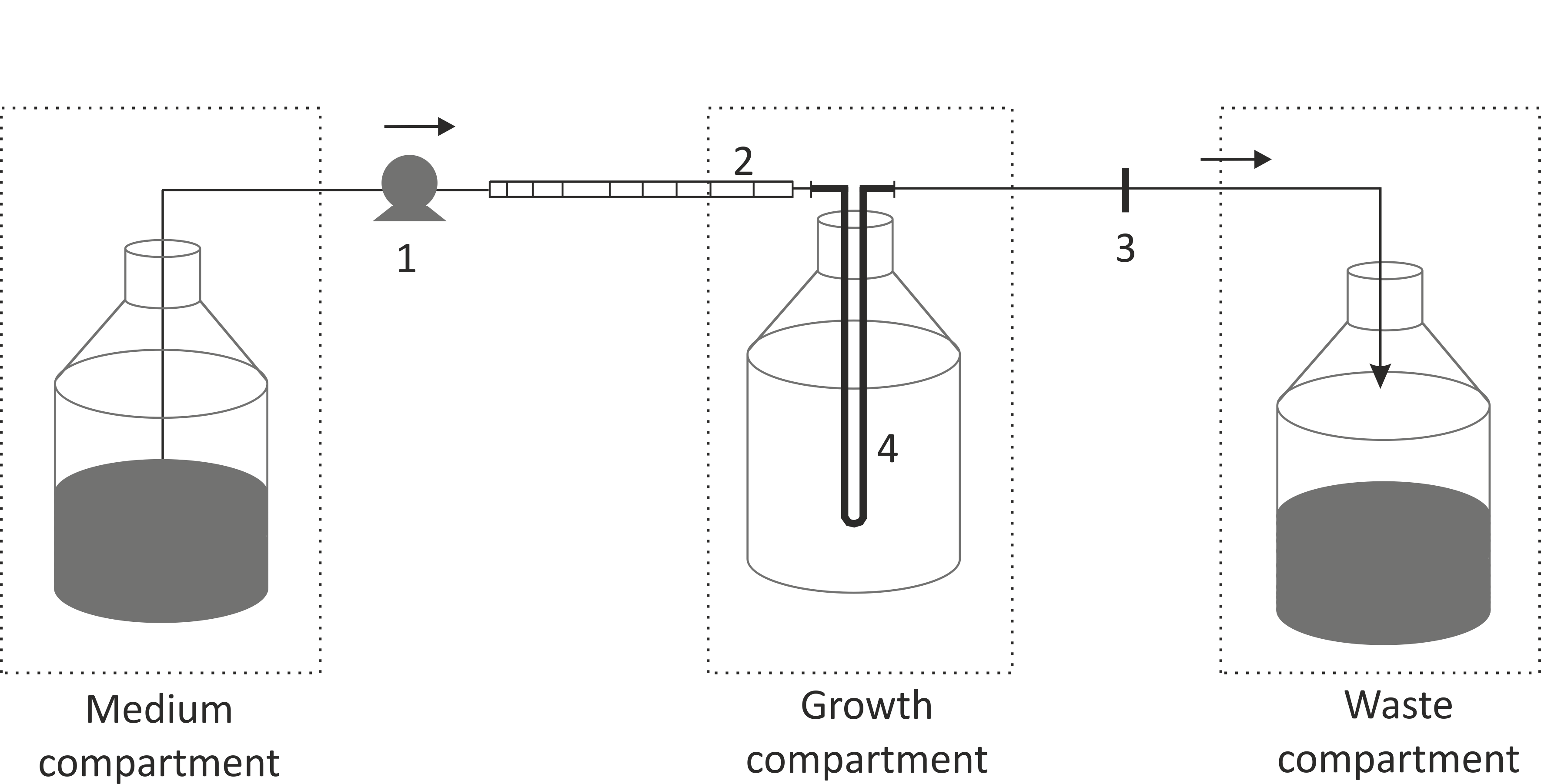


**Figure S1:** Schematic diagram of the tubular setup applied for biofilm biomass production.

(1) Peristaltic pump; (2) MASTERFLEX^®^ tube; (3) sampling port for aqueous phase outlet; (4) Silicone tube as biofilm substratum; The dotted lines highlight the individual compartments. The setup was kept in a water-bath and the temperature was set to 30°C.

**Figure S2**

**
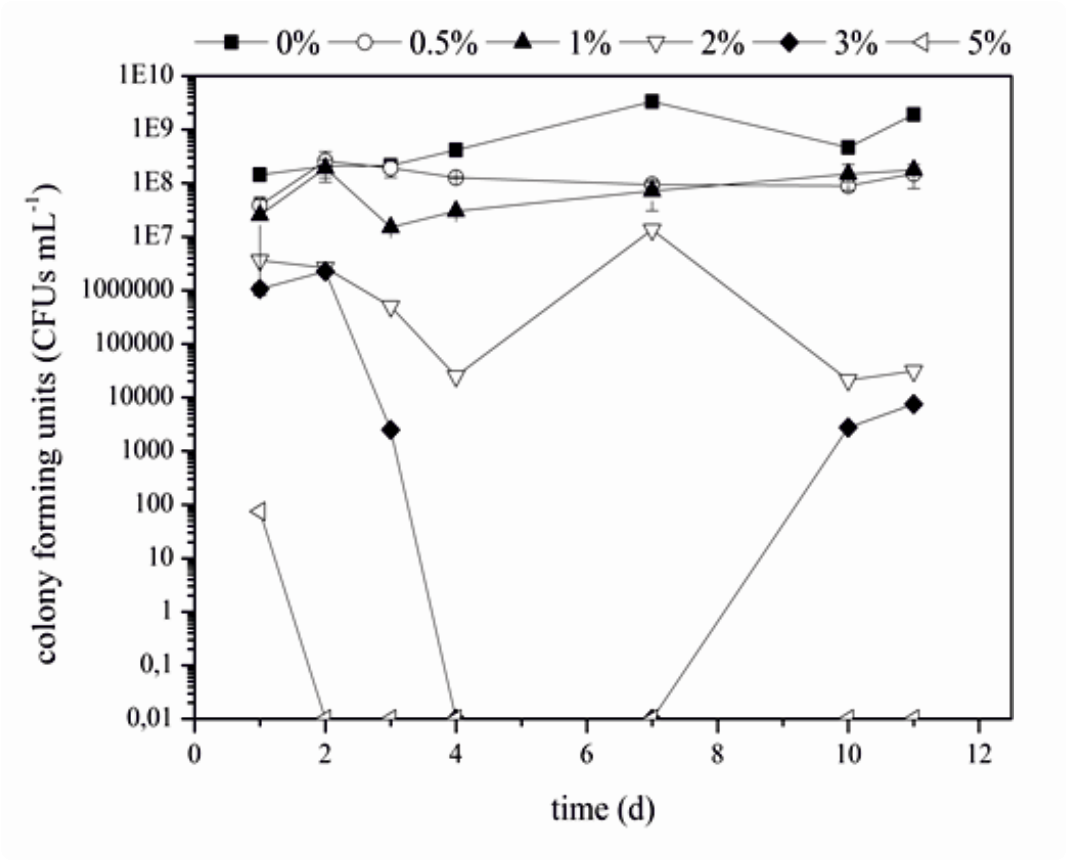
**

**Figure S2:** Survival of *P.* *taiwanensis* VLB120 grown in biofilms treated with different concentrations of butanol determined by colony forming units (CFU). Biofilms were grown in silicon tubes, constantly supplied with LB medium supplemented with 10 g L^-1^ of glucose. After 3 days of growth, different concentration of butanol was added to the feed solution (day 0 of the experiment) and subsequently colony forming units (CFU/mL) were counted. Respective samples were collected from the outlet of the silicone tube and plated on LB Agar plates after appropriate dilutions. The CFU values reflecting the number of detached cells from 0.004 m^2^ biofilm carrier surfaces (silicone tubing) operated at a flow rate of 50 µL min^-1^.

**Figure S3**

**
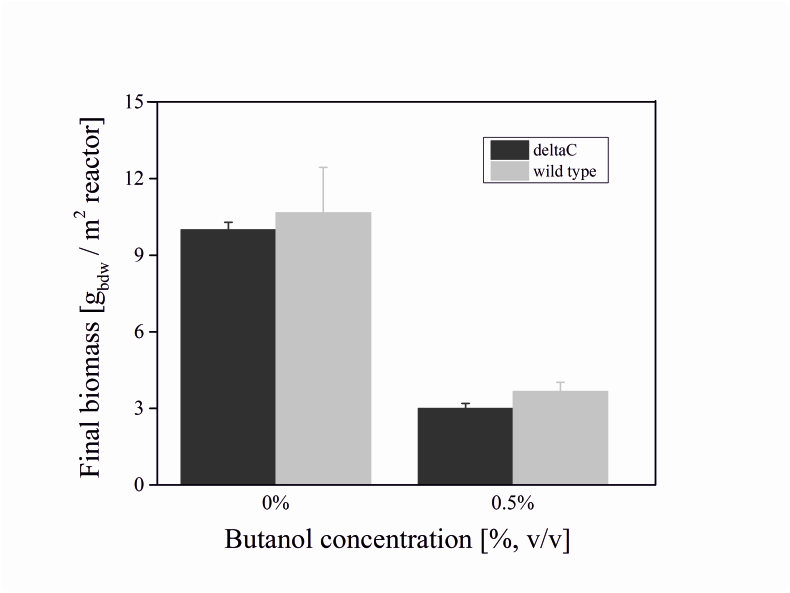
**

**Figure S3:** Comparison of butanol concentration dependent biomass yield of *P.* *taiwanensis* VLB120 and mutant strain *P.* *taiwanensis* VLB120∆C biofilms. Biofilm growth expressed as the amount of biomass produced on a given reactor surface in the presence of different butanol concentrations. Biofilm was cultivated for 2 days. Data presented here are mean values from 4 parallel experiments.

**Figure S4**

**
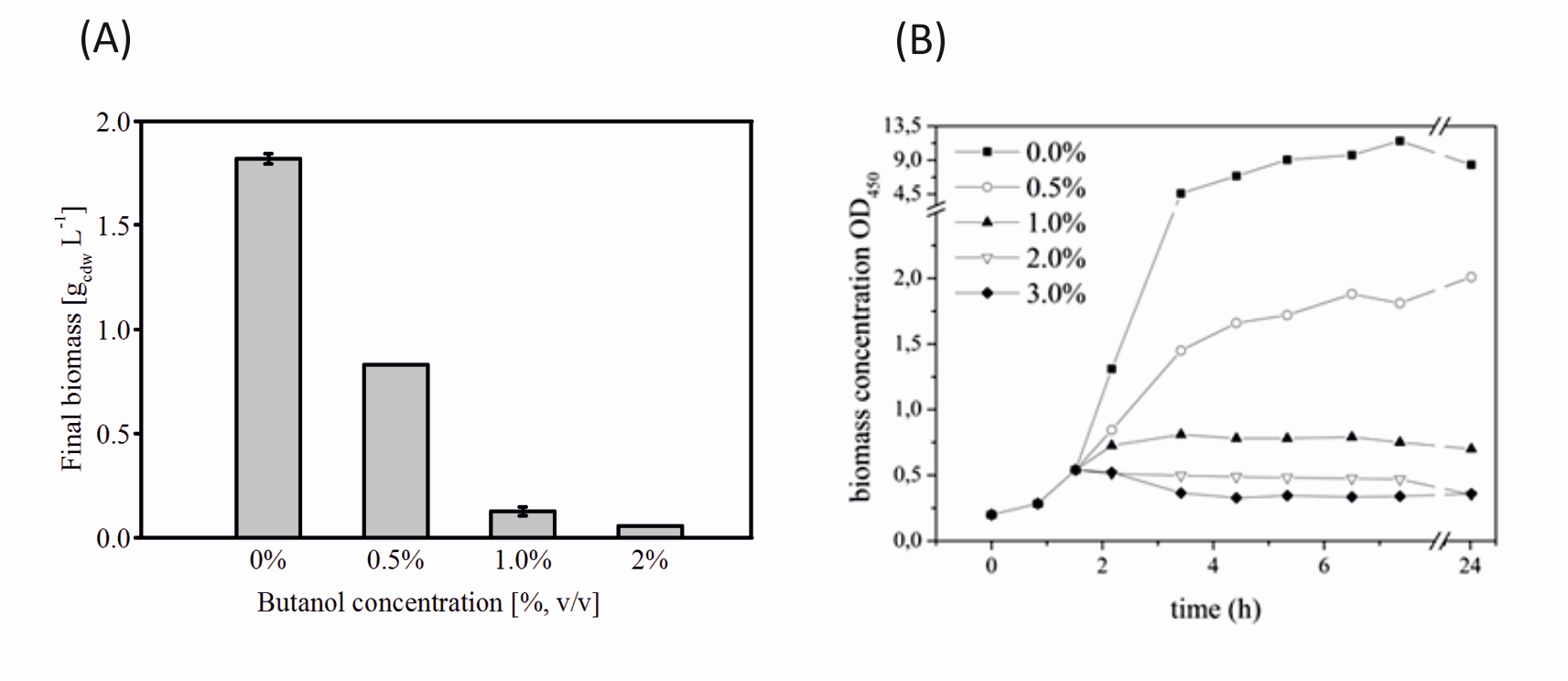
**

**Figure S4:** Butanol concentration dependent biomass yield of planktonically grown *P.* *taiwanensis* VLB120 and *P.* *taiwanensis* VLB120∆C. (A) - *P.* *taiwanensis* VLB120∆C growth expressed as the amount of final biomass density achieved in baffled shake flask (M9-minimal medium with 0.5% glucose) in the presence of different butanol concentrations. Cells were cultivated for 10 hours. (B) - *P.* *taiwanensis* VLB120 growth expressed as the amount of biomass density (OD_450nm_) achieved in baffled shake flask (LB medium with 1% glucose) in the presence of different butanol concentrations. Data presented here are mean values from 3 parallel experiments (biological triplicates).

**Table S1: Methods for EPS component analysis**

| **Compound** | **Assay** | **Standard** | **Reference** |
| --- | --- | --- | --- |
| Protein | Lowry | Bovine Serum Albumin | Lowry *et al.,* 1951 |
| Carbohydrate | Phenol sulfuric acid | D-Glucose | Dubois *et al.,* 1956 |
| Lipid | Sulfo-phospho-vanillin | Olive oil | Frings *et al.,* 1972 |
| Uronic acids | Meta-hydroxydiphenyl | D-glucuronic acid | Filisetti-Cozzi and Carpita 1991 |

**References**

1. Dubois, M., Gilles, K. A., Hamilton, J. K., Rebers, P. A., Smith, F. (1956) Colorimetric Method for Determination of Sugars and Related Substances. *Anal Chem*. **28**: 350–356
2. Filisetti-Cozzi, T.M., Carpita, N.C., (1991) Measurement of uronic acids without interference from neutral sugars. *Anal Biochem*. **197**:157-62.
3. Frings, C.S., Fendley, T.W., Dunn, R.T., Queen, C.A. (1972) Improved determination of total serum lipids by the sulfo-phospho-vanillin reaction. *Clin Chem*. **18**:673-674.
4. Lowry, O.H., Rosebrough, N.J., Farr, A.L., Randall, R.J. (1951) Protein measurement with the Folin phenol reagent. *J Biol Chem*. **193**:265-275.
